# Epigenetic Landscape and Regulation of Secondary Metabolite Accumulation Implicated in Anticancer and Nutraceuticals

**DOI:** 10.3390/epigenomes10040066

**Published:** 2026-10-12

**Authors:** Deekshitha Kosaraju, Viswanadha Naik Jarapala, Aparna Yeddala, Charumathi Polavarapu, Vasavi Singareddy, Sudhakar Reddy Palakolanu, Nese Sreenivasulu, Polavarapu Bilhan Kavi Kishor

**Affiliations:** 1 Independent Researcher, Dallas, TX 75201, USA; deekshithakosaraju0819@gmail.com; 2 Center for Plant Molecular Biology, Department of Genetics, Osmania University, Hyderabad 500007, India; jvnaik1@gmail.com; 3 MVSR Engineering College, Nadergul, Hyderabad 501510, India; aparnayeddala@gmail.com; 4 Acute Care Physical Therapist, St. Luke’s Health, 1717 US-59 Loop North, Livingston, TX 77351, USA; charu.physio@gmail.com; 5 International Crops Research Institute for the Semi-Arid Tropics (ICRISAT), Hyderabad 502324, India; singareddyvasavi@gmail.com (V.S.); sudhakarreddy.palakolanu@icrisat.org (S.R.P.); 6 International Rice Research Institute, DAPO Box 7777, Metro Manila 1301, Philippines; nesesreenivasulu7@gmail.com; 7 Department of Agriculture, Dr. RVR NRI Institute of Technology (Deemed to be University), Pothavarappadu, Vijayawada Rural 521212, India

**Keywords:** anticancer products, cell cultures, epigenetic factors, nutraceuticals

## Abstract

Plants are important sources for obtaining diverse secondary metabolites which have pharmaceutical importance. Out of multiple factors that modulate their accumulation in vitro and in vivo, epigenetic factors play critical roles, adding an extra layer of regulation. This review focuses on (a) dynamic changes that occur in DNA methylation, histone acetylation, and the role of small RNAs in transcriptional and post-transcriptional modulation that alter secondary metabolism in plants under abiotic stress and (b) the role of epigenetics in secondary metabolite accumulation in the plant cell cultures. Such epigenetic factors are manipulated by applying inhibitors of DNA methylation like 5-azacytidine or histone deacetylation such as trichostatin A in intact plants as well as in cultured plant cells and hairy roots for enhanced production. This article synthesizes current findings on epigenetic engineering for improved synthesis of anticancer compounds and nutraceuticals and lists out limitations and challenges that the pharmaceutical industry is facing for their large scale production and commercialization. Main conclusion: Through epigenetic changes such as DNA methylation, histone acetylation, and non-coding RNAs, plants dynamically fine-tune specific gene expressions to adjust to environmental stresses and mediate secondary metabolite accumulation as a chemical combat mechanism.

## 1. Introduction

Both primary and secondary metabolism are closely connected in plants. Primary metabolism provides the energy (ATP and NADPH) and the necessary building blocks to biosynthesize secondary metabolites that help in plant defense and function as signaling compounds [1]. Primary metabolic processes such as photosynthesis and glucose oxidation produce carbohydrates, amino acids, and fatty acids required for plant growth and development. Primary metabolism pathway molecules act as precursors, supply ATP, and NADPH for the biosynthesis of secondary metabolites like alkaloids, phenolic compounds, terpenoids, and others. Importantly, plants balance both carbon and nitrogen resources between primary and secondary metabolism. However, when faced with environmental stresses, they redirect metabolic flux from growth into secondary metabolite biosynthesis [2].

**Figure 1:**
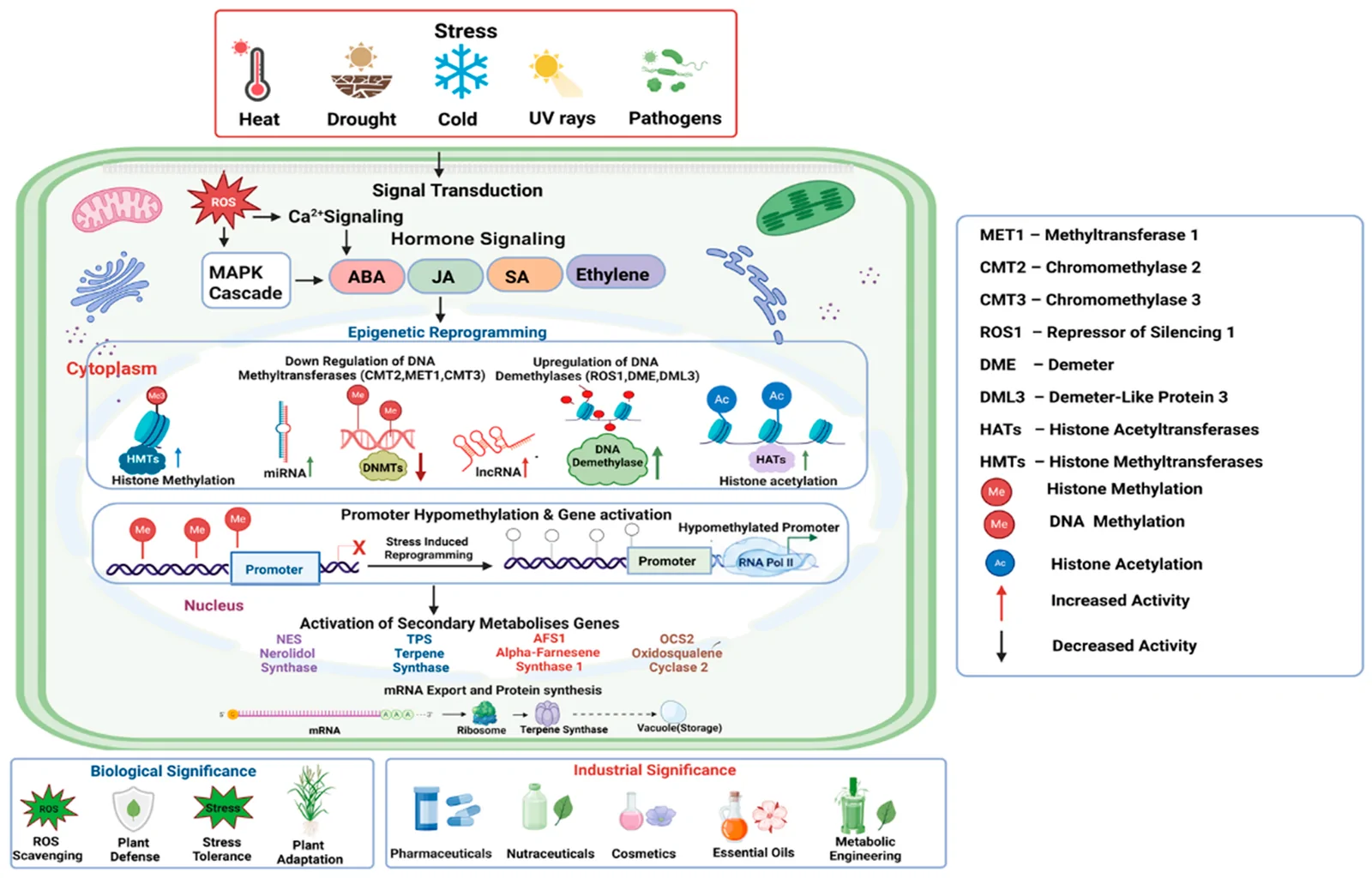
Stress-induced epigenetic reprogramming regulates secondary metabolite biosynthesis in plants. Environmental stresses, including heat, drought, salinity, cold, UV radiation, and pathogen/herbivore attack, are recognized by plasma membrane-associated receptors, triggering ROS production, MAPK and Ca^2+^ signaling, and phytohormone-mediated responses (ABA—abscisic acid, JA—jasmonic acid, SA—salicylic acid, ROS—reactive oxygen species, and ethylene). These signaling cascades coordinate in the nucleus to induce epigenetic reprogramming through reduced DNA methylation, active DNA demethylation, histone acetylation, histone methylation, chromatin remodeling, and non-coding RNA (miRNA and lncRNA)-mediated regulation, leading to promoter hypomethylation, chromatin relaxation, and recruitment of RNA polymerase. This leads to secondary metabolite biosynthetic genes, including NES—nerolidol synthase, TPS—terpene synthase, AFS—α-farnesene synthase, and OCS—ocimene synthase, are transcriptionally activated, followed by mRNA export and protein synthesis. The resulting increase in specialized metabolites contributes to improved antioxidant defense, stress tolerance, plant resilience, and the production of high-value natural products with pharmaceutical, nutraceutical, cosmetic, and industrial applications. Note: 1. Red downward arrow beside DNMTs: indicates decreased DNA methyltransferase activity. 2. Red upward arrow beside lncRNA: indicates increased lncRNA expression or activity. 3. Green upward arrows: indicate increased activity of the associated components. 4. Blue upward arrow beside HMTs: indicates increased histone methyltransferase activity and black arrows indicate the direction of signaling or regulatory processes.

**Figure 2:**
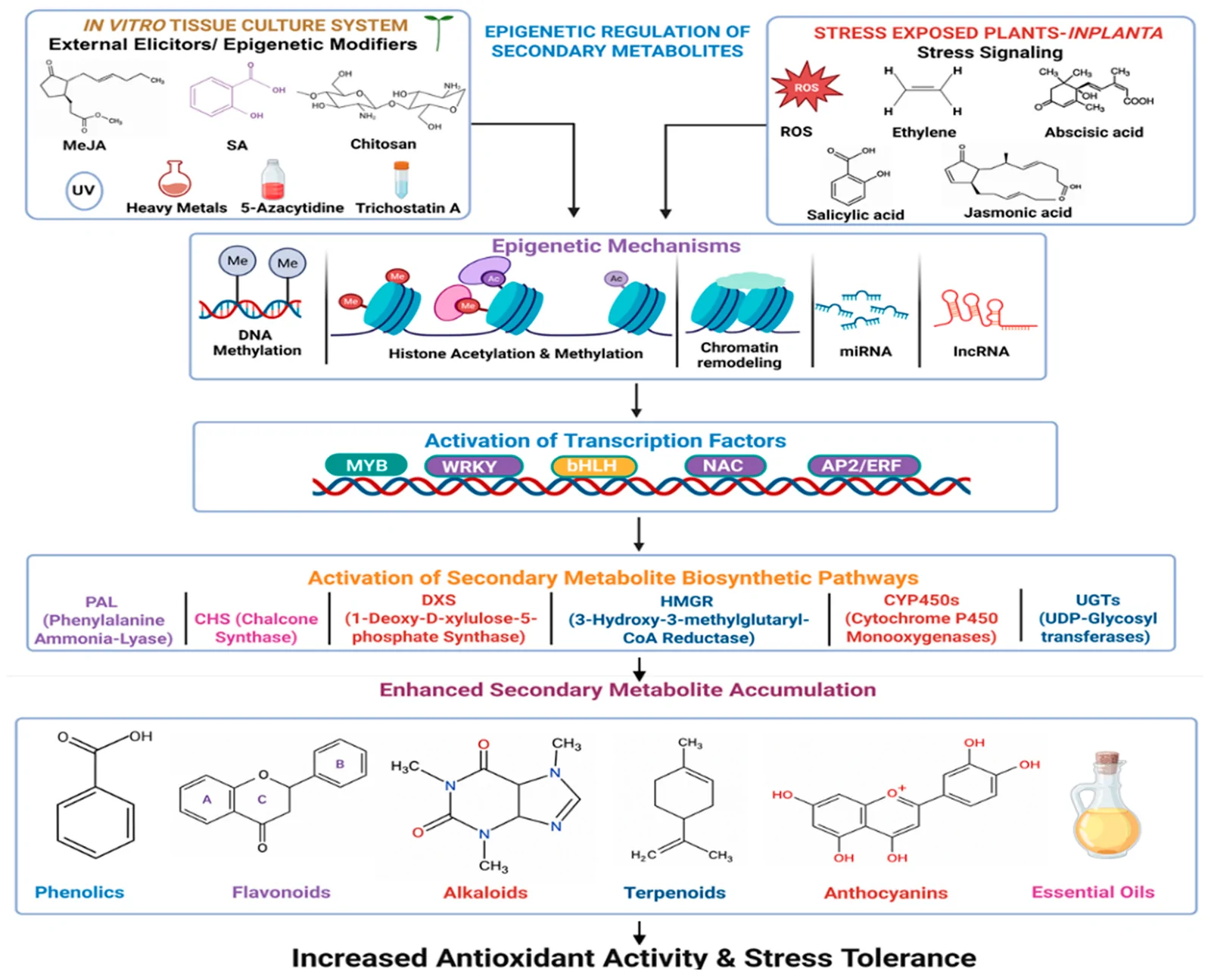
Integrated epigenetic regulation of secondary metabolite biosynthesis in in vitro tissue culture systems and stress-exposed plants. In vitro tissue culture systems employ external elicitors and epigenetic modifiers such as methyl jasmonate (MeJA), salicylic acid (SA), chitosan, ultraviolet (UV) radiation, heavy metals, 5-azacytidine (5-AzaC), and trichostatin A (TSA) to induce epigenetic reprogramming. In parallel, environmental stresses in *in planta* systems, including drought, salinity, heat, cold, UV radiation, and pathogen/herbivore attack, activate stress signaling pathways mediated by reactive oxygen species (ROS) and phytohormones (ABA—abscisic acid, JA—jasmonic acid, SA—salicylic acid, and ethylene). These distinct upstream stimuli collectively regulate common epigenetic mechanisms, including DNA methylation, histone acetylation, histone methylation, chromatin remodeling, and non-coding RNA (miRNA and lncRNA)-mediated regulation, leading to transcriptional activation of stress-responsive transcription factors (MYB—myeloblastosis transcription factor, WRKY—WRKY transcription factor, bHLH—Basic Helix–Loop–Helix transcription factor, NAC—NAM, ATAF1/ATAF2, and CUC2 transcription factor, and AP2/ERF—APETALA2/ethylene-responsive factor) and secondary metabolite biosynthetic genes such as PAL (phenylalanine ammonia-lyase), CHS (chalcone synthase), DXS (1-deoxy-D-xylulose-5-phosphate synthase), HMGR (3-hydroxy-3-methylglutaryl-CoA reductase), CYP450s (cytochrome P450 monooxygenases), and UGTs (UDP-glycosyltransferases). Coordinated activation of these pathways enhances the accumulation of diverse secondary metabolites, including phenolics, flavonoids, alkaloids, terpenoids, anthocyanins, and essential oils, thereby improving antioxidant capacity, stress tolerance, and adaptation.

Plants produce a wide variety of secondary metabolites with potential medical uses. Field-grown plants or cultured cells that are exposed to stressful conditions exhibit epigenetic modifications which may affect the biosynthesis of secondary metabolites implicated in defense and stress adaptation. To survive and adapt themselves to stressful conditions, plants reroute both carbon and nitrogen resources for the biosynthesis of secondary metabolites, thus undergoing complex metabolic reprogramming. Such metabolic processes are tightly regulated by epigenetic factors such as DNA methylation, histone modification, chromatin remodeling, RNA interference (RNAi), or non-coding RNAs which control the accessibility of DNA to alter post-transcriptional activities leading to gene expressions and stimulation of secondary metabolite biosynthetic pathways. Secondary metabolite biosynthetic gene clusters (BGCs) enriched with transposons, the histone variant H2A.Z, and the histone modification H3K27me3 tightly coordinate gene expressions under stressful environments and facilitate accumulation. There are three epigenetic mechanisms that govern plant growth and development, which include DNA methylation, histone modification, and non-coding RNAs. Stress-induced epigenetic reprogramming regulates secondary metabolite biosynthesis in plants, as shown in Figure 1. Additionally, an integrated epigenetic regulation of secondary metabolite biosynthesis in in vitro tissue culture systems and stress-exposed plants is schematically represented in Figure 2.

Accumulation of many pharmaceutically important compounds in plants occurs in ultra-low or trace quantities (e.g., artemisinin, atropine, camptothecin, codeine, morphine, paclitaxel, podophyllotoxin, vincristine, vinblastine, etc.). Further, our valuable plant resources are dwindling year after year due to deforestation [3]. Consistent production of secondary metabolites from plants is also a serious concern due to seasonal variations and environmental issues like pathogen contaminations, severe drought, metal, and low and high temperature stresses. Further, there is a growing demand for plant-based secondary metabolites, which necessitates the use and exploration of green biotechnological tools to produce genetically modified plant cells and hairy roots for yield optimization and product enrichment and stability [4,5,6]. In plant cell cultures, production occurs under controlled laboratory or bioreactor conditions and hence a consistent and reliable supply of secondary metabolites with uniform quality is ensured. Metabolites isolated from plant cell cultures are free from heavy metals, pesticides, and pathogen contaminations, hence pharmaceutical industries prefer them. Many rare secondary metabolites with complex chemical structures cannot be synthesized chemically. However, plant suspensions and hairy roots produce such rare and complex compounds, albeit to a lesser extent. To face biotic and abiotic stresses, and to counteract unfavorable conditions, plants unlock many defensive mechanisms at the physiological and molecular level [5,7] and biosynthesize secondary metabolites like alkaloids, phenylpropanoids, sulfur-containing compounds, terpenoids, and nutraceuticals [8,9].

Plant-derived compounds supply nearly 60% of approved anticancer compounds globally. Clinically significant plant-derived anticancer compounds include paclitaxel derived from *Taxus* species, vincristine and vinblastine isolated from *Catharanthus roseus*, camptothecin from *Camptotheca acuminata*, podophyllotoxin from *Podophyllum peltatum*, harringtonin from *Cephalotaxus*, and others. Therefore, their regulation by epigenetic modification is vital for us. Nutraceutical compounds are food-derived substances which provide health benefits and bridge the gap between food and pharmaceutical medicine; common examples include antioxidants, dietary fibers, omega-3-fatty acids, minerals, polyphenols, prebiotics, probiotics, and vitamins [10]. They are available in whole foods and commercially marketed in the form of capsules, tablets, or functional foods. Regular intake of nutraceuticals helps in lowering the risk factors associated with cancer, heart ailments, obesity, and type 2 diabetes. Nutraceuticals neutralize free radicals, improve nutrient absorption and immune function, maintain intestinal health and cognitive and mental well-being, and have anti-inflammatory action [11]. Epigenetic mechanisms in plants include DNA methylation, histone modifications, chromatin remodeling, and non-coding RNAs, which have led to a better understanding and expanded the potential for improving secondary metabolite accumulation. Epigenetic changes are heritable and non-heritable and their regulation plays a vital role in plant adaptation to environmental stresses. Epigenetic changes are critical for plant development, yield, nutritional quality, and acclimation to environmental stresses [12].

Berberine from *Coptis japonica*, ginsenosides from *Panax ginseng*, paclitaxel from *Taxus chinensis*, podophyllotoxin from *Podophyllum hexandrum*, rosmarinic acid from *Coleus blumei*, and shikonin from *Lithospermum erythrorhizon* cell cultures are being commercially produced using plant cell cultures [13,14,15]. In cell cultures, transcriptomic and metabolomic studies have helped to identify the biosynthetic pathways and the genes associated in the biosynthesis [16,17]. However, industrial production of specialized compounds has been achieved only in a few species. Epigenetic mechanisms regulating the pathways are highly complex and so is the production of secondary metabolites under abiotic stress exposure of plants and epigenetic modifications and their impact on gene expressions in increasing secondary metabolite accumulation in cell cultures. The review examines how epigenetic modifications impact the accumulation of secondary metabolites while providing a perspective on the multiple layers of gene expression regulation. The manuscript offers a compelling and relevant exploration of the epigenetic modifications that occur both in vivo and in vitro especially in anticancer and also in nutraceutical producing plants/cell cultures. This review discusses and covers the scope of chemical intervention for demethylation and how specific agents bypass genetic engineering to enhance yields.

## 2. Epigenetic Changes Under Stress Reprograms Secondary Metabolism

Exposure of plants or plant cells to water stress, high and low temperatures, and also biotic elicitor-induced patterns is imperative for primary metabolite reprogramming and to channel the precursors necessary for the biosynthesis of secondary metabolites [18]. Under normal conditions, primary metabolites such as carbohydrates, amino acids, and others are utilized for growth and development. However, during stress conditions, the carbon flux slows down, the pentose phosphate pathway is stimulated to generate energy (NADPH), and metabolites are redirected for the biosynthesis of secondary metabolites. Epigenetic regulators interact with plant hormone signaling pathways [jasmonic acid (JA), salicylic acid (SA), and abscisic acid (ABA)] to modulate stress responses. Both hormonal and epigenetic signals stimulate TFs, featuring mainly *WRKY*, *MYB*, and *bHLH*, which bind to the biosynthetic genes and direct biosynthetic pathway (the shikimate pathway that synthesizes alkaloids, flavonoids, and phenylpropanoids; the methylerythritol phosphate and mevalonate pathways synthesize essential oils and terpenoids; and the TCA cycle helps to synthesize nitrogen-containing alkaloids, cyanogenic glycosides, and glucosinolates) [19]. Collectively, studies indicate that primary metabolism is redirected for the synthesis of secondary metabolites during stress; notably, sugar-sensing networks, where trehalose-6-phosphate (T6P), hexokinase, and SnRK1 kinases coordinate the energy status of the cells with stress responses and allocation of resources to the specific metabolism [20]. The sensors modulate primary pathways and key enzymes in the secondary metabolic pathways under resource-limited conditions. Stress-induced DNA methylation changes can suppress or trigger the gene activities associated with phenylpropanoid, terpenoid, alkaloid, and other biosynthetic pathways. For instance, water and salinity stresses cause promoter demethylation of proline biosynthesis genes such as *pyrroline 5-carboxylate synthase 1* (*P5CS1*) in watermelon [21] and capsaicinoid and flavonoid biosynthesis in capsicum, respectively, contributing to osmotic adjustment and thereby improving stress tolerance and antioxidant capacity [22]. On the other hand, DNA demethylation is catalyzed by enzymes like REPRESSOR OF SILENCING 1 (ROS1) and DEMETER (DME), which play a key role in metabolic reprogramming. DNA demethylation results in the stimulation of stress-responsive genes, including antioxidant pathways [23].

Dynamic changes in DNA and histones allow plants to fine-tune the expression of genes associated with growth, stress responses, and metabolic adjustments. Stress-induced DNA methylations or histone acetylations either can suppress or stimulate genes involved in specific secondary metabolite biosynthesis pathways [24]. At the same time, stress enhances histone acetylations at promoter regions of key genes involved in secondary metabolite synthesis [25]. In *A. thaliana*, drought stress results in hyperacetylation of histones H3 and H4 at the promoters of *PHENYLALANINE AMMONIA-LYASE* (*PAL*), *CHALCONE SYNTHASE* (*CHS*), and *FLAVANONE 3-HYDROXYLASE* (*F3H*) genes and stimulation of flavonoid biosynthesis [9]. Histone methylations can either activate or repress transcription depending on the degree of methylation. While marks like H3K4me3 and H3K36me3 trigger gene expressions, H3K27me3 and H3K9me2 suppress gene activity. Stress-induced reprogramming of these epigenetic changes regulates the genes implicated in secondary metabolism. For example, cold stress decreased H3K27me3 at anthocyanin biosynthesis gene loci, resulting in enhanced anthocyanin [26]. Cold stress- and epigenetics-regulated accumulation of flavonoids and terpenoids was reported [27]. DNA methylation governs the saponin accumulation in *Eleutherococcus senticosus* [28]. Additionally, epigenetic regulation of alkaloid, anthocyanin, and flavonoid biosynthesis were reviewed in higher plants [29,30,31].

Besides DNA methylations and histone acetylations, small, non-coding RNA (ncRNA) molecules like microRNAs (miRNAs), small interfering RNAs (siRNAs), and long non-coding RNAs (lncRNAs) guide proteins to target specific gene sequences and thus play a critical role in post-transcriptional gene silencing by degrading native mRNA, thereby ensure modulation of growth, development, and stress responses [32]. Thus, miRNAs, siRNAs and lncRNAs regulate chromatin states, DNA methylation, and histone modifications, which affect the expression of genes associated with environmental responses [33]. While stress upregulates secondary metabolism, it also downregulates primary metabolite biosynthetic pathways. Chromatin remodelers like SWItch/Sucrose Non-Fermentable (SWI/SNF) complexes and histone modifiers respond to signals by regulating TFs like WRKYs and MYBs. These TFs fine-tune metabolic reprogramming, thereby improving the accumulation of secondary metabolites. Both small RNAs and lncRNAs bridge early stress signaling with metabolic reprogramming [33]. Stress-induced miRNAs such as miR156, miR172, and miR319 target TFs involved in the regulation of secondary metabolic pathways and stress tolerance [34]. miRNAs can alter epigenetic changes by influencing DNA methyltransferases [35]. LncRNAs (>200 nucleotides) act as scaffolds for chromatin-modifying complexes and guide histone methyltransferases or demethylases to target genes. During drought and salinity stresses, lncRNAs modulate secondary metabolism by controlling TFs and biosynthetic pathway genes [36]. Mobile siRNAs and miRNAs noticed in phloem sap of plants infer that they play a role in fundamental stress responses [37]. Stress-induced ncRNAs are critical in plant stress memory and modulate gene expression at transcriptional and post-transcriptional levels and pass on epigenetic changes that enable plants to respond to abiotic stresses [38]. The growing body of evidence indicates that ncRNAs act as key players in plant epigenetic regulation linking environmental stress and secondary metabolism.

## 3. Epigenetic Regulation of In Vivo and In Vitro Secondary Metabolite Accumulation

In the case of tissue culture, isolation of explants coupled with in vitro conditions stimulate unprogrammed but perpetual epigenetic changes which are not readily reversible [9,39]. Additionally, explant source, stress memory, and secondary metabolite synthesis are interrelated in plant tissue culture. While initial tissue determines the epigenetic changes to occur, short- and long-term stress imprints modulate the biosynthetic pathways and stimulate the secondary metabolite synthesis. Initial explants like immature embryo, leaf, meristem, or root determine the path that a cell takes over a period of time [40]. Production of secondary metabolites is organ-specific in field-grown plants [41]. Callus, suspensions, and organ cultures derived from specific tissues retain memory of their tissue of origin [42]. Hence, explants that produce higher quantities of secondary metabolites are preferred for culturing in vitro. However, maintaining dedifferentiated cells in vitro for a prolonged period leads to the decline or total loss of specialized secondary metabolite production, since the pathways generally rely on some level of cellular differentiation, organized tissue structures, and specialized secretory compartments for gene expressions and subsequent accumulation [43]. The phenomenon of epigenetics is transmitted to the next generation in some cases [44]. Hence, understanding global methylation, demethylation patterns, chromatin remodeling, histone modifications, and RNA interference which regulate biosynthetic pathways open up the possibility of reversing the phenomenon of loss of potential to accumulate high-value secondary metabolites in cultured cells. Among the strategies to improve secondary metabolites, targeting biosynthetic genes such as *3-hydroxy-3-methylglutaryl-CoA reductase* (*HMGR*) for terpenoid synthesis, *phenylalanine ammonia-lyase* (*PAL*) for phenolic synthesis [9] and transcription factors (TFs) like *bHLH*, and *MYB* that control anthocyanin and flavonoid synthesis and also *octadecanoid-responsive Catharanthus AP2-domain* (*ORCA*) that regulates terpenoid and indole alkaloid biosynthesis are crucial [29,45]. Some of the epigenetic modifications and their influence on secondary metabolite biosynthesis are described below.

Epigenetic research remains mostly underexplored for critical medicinal plants like *Catharanthus roseus*, *Cinchona officinalis*, *Pausinystalia johimbe*, *Rauwolfia serpentina*, *Strychnos nux-vomica*, *Taxus brevifolia*, and *Withania somnifera*, where epigenetic studies are lagging [46]. What we still need to know is whether variations in DNA methylation profiles and histone modifications control the developmental and environmental regulation of the specialized metabolites in a tissue-specific manner in diverse medicinal plants. CRISPR/Cas9-based epigenome editing tools currently exist, but their use in regulating specific metabolic pathways remains limited [47]. In *Catharanthus roseus* [46] and other medicinal plants, low endogenous yields of secondary metabolites are an obstacle for their commercialization. The epigenetics governing the expression of biosynthetic pathway gene clusters remained largely unmapped or entirely missing in comparison with model crops. Therefore, it is vital for us to develop platforms that can precisely edit the epigenome in critical medicinal plants used in anticancer and nutraceutical industry for the optimization of specialized metabolites in cultured cells/hairy roots and field-grown plants.

## 4. Anticancer Molecules and Their Regulation by Epigenetics

**Figure 3:**
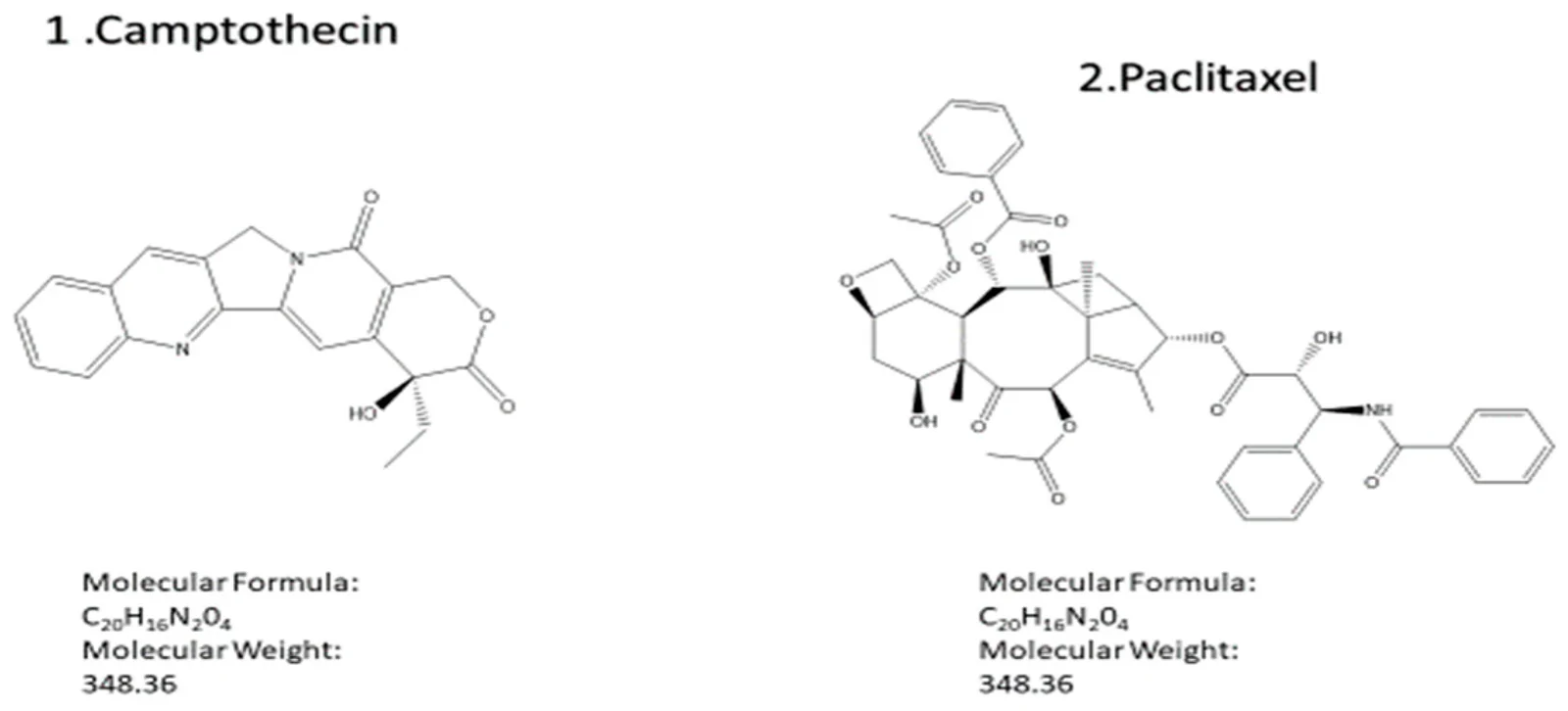

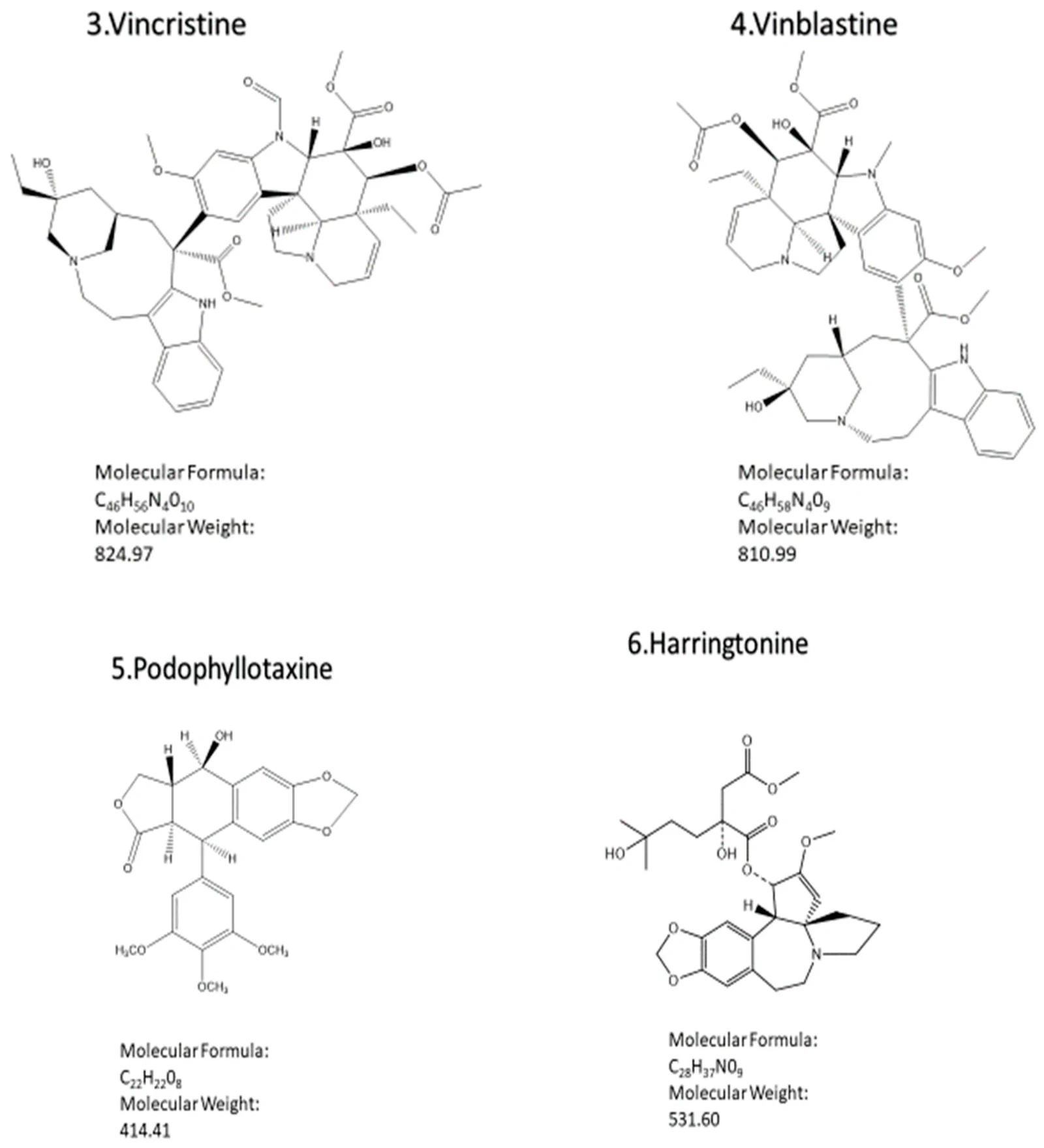
Chemical structures of plant-derived anticancer compounds approved by FDA.

Several secondary metabolites derived from plants have been shown to act against cancer. However, in this review, only those plant-derived molecules approved by the FDA for therapy (except hypercin, which is an important agent used in photodynamic therapy) are considered for epigenetic regulation. Chemical structures of some important plant-derived anticancer compounds approved by the FDA are shown in Figure 3.

### 4.1. Camptothecin Derived from Camptotheca

*Camptotheca acuminata* produces monoterpene indole alkaloids (MIAs), importantly camptothecin. Camptothecin is not directly used as a chemotherapy drug since its solubility is poor and toxicity is high. However, its derivatives such as topotecan and irinotecan are prescribed widely for treating cervical, colorectal, lung, and ovarian cancer types [48]. DNA methylations largely govern monoterpene indole alkaloid (MIA) accumulation in *C. acuminata* [49]. Accumulation of camptothecin biosynthesis is negatively modulated by light. Shading or low-light intensities promote camptothecin accumulation in *C. acuminata* [50]. The Light-Mediated camptothecin Biosynthesis Factor (*CaLMF*) is a bZIP-type TF that suppresses genes such as *CaTDC1*, *CaG80*, *CaCYC1*, and *Ca7-DLS1*. Under light conditions, *CaLMF* expression enhances and inhibits biosynthetic pathway genes and camptothecin accumulation. However, under shading, *CaLMF* expression is downregulated and camptothecin production is enhanced. Heterologous expression of *CrLAMT* and *O*-methyltransferase from *C. roseus* in *C. acuminata* activated the methylated intermediate pathway in *C. acuminata*, resulting in the conversion of loganic acid to loganin. This approach led to enhanced accumulation of camptothecin in hairy roots by 2.23-fold and 1.78-fold in transgenic material [51]. This strategy to improve high-value camptothecin yield can be further exploited to produce the alkaloid in large quantities. Zhang et al. [52] evaluated the effects of NaCl stress in *C. acuminata* in vitro. The authors used Inter-Simple Sequence Repeat (ISSR) markers and traced the relation between salt stress and genomic DNA methylation. The experiments revealed tissue-specific hyper/hypomethylation which correlates with changes in cell proliferation and accumulation of campthothecin and 10-hydroxycamptothecin [52]. Demethylation events were detected near stress-responsive transposable elements and metabolic genes, upregulating MIA genes like *G10H* and *SLS*. The results indicate that the overall epigenetic response is systemic. Demethylation events trigger before the upregulation of biosynthetic genes and the subsequent accumulation of camptothecin. To achieve high camptothecin levels, it is necessary to map DNA methylation and histone modification patterns across the genome and use the data thus generated for metabolic engineering of specific genes. We also need to identify how chromatin remodeling affects the biosynthetic pathway genes like *TRYPTOPHAN DECARBOXYLASE* (*TDC*) and *GERANIOL 10-HYDROXYLASE* (*G10H*) and secondary metabolite accumulation. To date, no specific miRNAs involved in camptothecin biosynthesis have been generated.

#### Camptothecin Derived from *Ophiorhiza*

Establishing a causal relationship between DNA methylation or histone modification, gene expression, and secondary metabolite accumulation needs direct functional validation locus-specific epigenetic editing (dCas9-directed modifiers) or using chemical epigenetic inhibitors to modify expression and metabolic flux. Otherwise, observed patterns of secondary metabolite accumulation largely remain correlative. However, changes in methylation pattern and metabolite yield under environmental stress may match. *Ophiorhiza pumila* and other species produce camptothecin like that of *Camptotheca acuminata*, but via divergent biosynthetic pathways. Treating root cultures of *Ophiorhiza* with DNA methylation inhibitors triggered biosynthetic genes that were suppressed and also enhanced the camptothecin accumulation [45,53]. Gene body methylation dynamics fine-tune the transcription of biosynthetic pathway genes activating spatial-specific accumulation of camptothecin, primarily in the roots [54]. Camptothecin accumulation is partly causal via chemical modifiers, but mostly correlative in intact plants like *C. acuminata*, and *O. pumila*, DNA methylation and histone acetylation levels correlate well with the expression of tryptophan decarboxylase (TDC) [55]. Causality has been demonstrated in attenuated endophytic fungal systems and plant tissue cultures, where 5-azacytidine directly prevents gene silencing and restores camptothecin accumulation [56]. The transcription factors (*OpWRKY2*, *OpWRKY6*, and *OpNAC1*) are separated from that of epigenetic regulation. DNA or histone modifications have not been demonstrated clearly in these TFs or in their target genes. There is no evidence, however, that DNA methyltransferase inhibitors can upregulate camptothecin accumulation in *O. pumila*. No locus-specific methylations are reported so far. The TF *OpWRKY2* positively modulates camptothecin in *Ophiorhiza pumila* and enhances camptothecin production [57]. Likewise, *OpNAC1* TF serves as a negative regulator and suppresses *loganic acid O-methyltransferase* (*OpLAMT1*) expression. This intermediate step is necessary for the accumulation of loganin as well as camptothecin. Since *OpLAMT1* is suppressed, the pathway flux which is necessary for camptothecin production is decreased [58]. Additionally, there is no established literature which demonstrates that gene-body methylation directly fine-tunes camptothecin accumulation in *O. pumila*. Spatial control of camptothecin accumulation is controlled by localized *OpWRKY2* [59]. The highest accumulation of camptothecin was observed in roots and stems and lower levels in leaves, indicating its spatial expression of relevant genes. Studies on UV autofluorescence indicate that camptothecin accumulates in the vacuoles of hairy roots and moves to other cells through concentration-gradient-dependent mechanism [54]. Improved camptothecin using CRISPR/Cas9 has been achieved in transformed hairy roots, which is described in a separate section. A total of 78 *OpERF* genes were detected in *O. pumila*. Among them, *OpCBR1* exhibits dual roles in regulating camptothecin biosynthesis and hairy root growth [60]. Overexpression of *OpCBR1* inhibited camptothecin synthesis, but boosted root growth. Knockouts promoted higher camptothecin content, but suppressed root growth. The authors noticed that *OpCBR1* represses *iridoid oxidase* (*OpIO*) promoter to decrease camptothecin levels but, however, upregulates auxin- and cytokinin-related genes to promote biomass [60]. However, this has not been tested on an industrial/biotechnological scale.

### 4.2. Vinblastine and Vincristine

*Catharanthus roseus* produces several terpenoid indole alkaloids (TIAs) that have therapeutic value. Existing evidence indicates that DNA methylation regulates tissue-specific, developmental, and environmental accumulation of monoterpenoid indole alkaloids (MIAs) in *C. roseus*. Multi-omics approaches establish that organ-specific MIA accumulation occur simultaneously with differentially methylated regions at structural biosynthetic genes along with upstream TFs like *bHLH* and *AP2/ERF* families [61]. Therefore, it is an association between methylation levels and expression of biosynthetic genes and MIA accumulation. In *C. roseus*, baseline methylation is approximately 40% to 55% for CG, 30% to 40% for CHG, and decreases to 5 to 10% for CHH contexts [61]. In suspensions or hairy roots, methylation in the promoter regions of terpenoid indole alkaloid (TIA) pathway genes increases up to 20% to 45% in CG and CHG contents. Treating with 20 µM 5-azacytidine, caused drop in genomic 5-methylcytosine levels from 0.65% to 0.33%. Reduction in methylation levels corresponds with the high transcript levels of the *decetylvindoline-4-O-acetyl transferase* (*DAT*) gene, which improves the synthesis of monoterpene indole alkaloids in the leaf [62]. The study indicates that *DAT* gene expression may be controlled by differential DNA methylation in the promoter and coding regions. Further, the study suggests that 5-azacytidine holds potential to modulate the expression of the *DAT* gene, resulting in higher vindoline biosynthesis. While genes implicated in vindoline alkaloid production are active in young leaves, they are suppressed due to methylation in root tissues [61]. Promoter regions of *STR* and *SLS* genes show high cytosine methylation under control conditions. Kumari et al. [63] investigated selection of *Catharanthus roseus* cultivar Nirmal (wild type) plants to salt stress and their correlation with targeted demethylation controlled by the single mutants like *lli* (leafless inflorescence), *egd* (evergreen dwarf), and *ill* (irregular leaf lamina) and double mutants displaying salt tolerance. While cytosines in CG contexts were 53% methylated, cytosines in CHH and CHG sites (where H = A, T, or C) contexts were 92% demethylated in the pleiotropic mutants [63]. The affected genes *LLI*, *EGD*, and *ILL* function as positive regulators for epigenetic cytosine methylation, bringing about salt stress tolerance and morphological characters in *C. roseus* [63]. In another study by Duge de Bernonville et al. [61], Whole-Genome Bisulfite Sequencing (WGBS) together with RNA-seq was used to identify differentially methylated and expressed genes among diverse samples [61]. They noticed that tissue-specific gene expression was associated with specific methylation depending on cytosine contexts. Some genes from the MIA pathway were differentially expressed and methylated with a corresponding MIA accumulation [61]. The findings indicated that TFs were influenced by alterations in DNA methylation. Overall, the experiments support an epigenetic regulation of specialized metabolism and likely targeting TFs which may modulate genes involved in the secondary metabolites.

Expression of *desacetoxyvindoline 4-hydroxylase* (*D4H*) gene involved in vindoline biosynthesis is transcriptionally blocked in cell suspensions, and therefore vindoline is not accumulated. The gene *D4H* is physically intact, but its lack of expression is attributed to lack of proper tissue differentiation (requires leaf epidermis, laticifer, and idioblast cells) [64]. However, it is not established if it is due to dedifferentiation-induced hypermethylation in *C. roseus* suspensions. Vindoline formation in shoot cultures of *C. roseus* is synchronously activated with morphogenesis [65]. *DAT* methylation during dedifferentiation was demonstrated to be a correlation for the loss of vindoline accumulation initially [64]. Experiments, however, reveal that DNA methylation states directly regulate *DAT* transcription in *C. roseus*. The loss of vindoline during dedifferentiation is multi-layered. Loss of vindoline biosynthesis in suspension cultures is firstly due to *DAT* gene silencing and then because of disruption of tissue architecture, which is required to complete the pathway. WGBS and tissue-specific profiling established a correlation between hyper/hypomethylation levels and the tissue specific loss or presence of vindoline pathway transcripts in intact plants, but in suspensions it is not directly demonstrated. The role of epigenetic changes and specific miRNAs that target anticancer compounds such as tetracyclic diterpenoids, vinca alkaloids, and aryltetralin lignin lactone are discussed in Table 1.

### 4.3. Harringtonine

Buds and leaves from 10-year-old trees of *Cephalotaxus harringtonia* produce the highest amounts of harringtonine and homoharringtonine, two important alkaloids. High accumulation in the buds and leaves indicates tissue-specific allocation of these alkaloids for carrying out specific physiological functions. Harringtonine measuring up to 240.9 mg/g dry weight and 16.9 mg/g dry weight of homoharringtonine were detected in *C. harringtonia* [77]. However, no DNA methylation and histone acetylation studies that aid in accumulation of harringtonine and homoharringtonine have been reported so far in vivo or in vitro. Therefore, scope exists for improvement of harringtonine using 5-azacytidine or other molecular methods.

### 4.4. Podophyllotoxin

*Podophyllum peltatum* and *P. hexandrum* produce podophyllotoxin, a potent cytotoxic lignan. Since podophyllotoxin itself is too toxic for human use, its semi-synthetic derivatives etoposide and teniposide are used as anticancer drugs [78]. Chromatin-level adjustments were observed due to environmental stress conditions [78]. Evidence for the accumulation of podophyllotoxin is however largely correlative. Further, a causal relationship mapping to an epigenetic change and the podophyllotoxin biosynthetic pathway genes have not been established clearly.

### 4.5. Paclitaxel

Dynamics of the initial phases of *de novo* RNA-directed DNA methylation (RdDM) in plant cells was investigated [79]. In old *Taxus* cell lines, the loss of paclitaxel production is due to DNA hypermethylation signatures in specific gene promoters. A comparative study of two cell lines, one 10-year-old with low productivity and the second with high productivity, demonstrated differences in growth and taxane accumulation and expression levels of taxane biosynthetic genes. The results indicate an altered pathway regulation and the *baccatin III: amino acid transferase* gene (*BAPT*) is active in flux-limiting step [66]. The methylation pattern of both the cell lines by bisulfite sequencing revealed high rates of CHH methylated cytosines on the core promoter, which has been identified as Y-patch promoter element in 10-year-old cell culture. The gene core exhibits 26.31% higher CHH methylation in older cultures than new lines, resulting in a gradual loss of paclitaxel yield over a period of 10 years. This implies a possible correlation between the loss of paclitaxel accumulation and DNA methylation at the promoters of biosynthetic pathway genes. The taxane accumulation peaked at the 24th day, and the old cell lines showed 2.48 mg L^−1^, and new cell culture 5.36 mg L^−1^ [66]. In another experiment, in 14-year-old *Taxus* cell lines, hypermethylation accumulated in the promoters of *taxadiene synthase* (*TXS*) and the *debenzoyl taxane 2a-O-benzoyltransferase* (*DBTNBT*) genes. *DBTNBT* promoter showed a 200 bp regulatory region before transcription start site with methylation accumulation. In old cell lines, the CHH cytosines are methylated within taxane-biosynthetic promoters; however, *GGPPS* genes are not methylated due to old age [80]. These experiments are an indication that in old suspensions or callus cultures, accumulation of secondary metabolites declines due to hypermethylation. Escrich et al. [67] observed that *DBTNBT* gene promoter has over 90% methylation in CG and CHG in both new and old cell cultures. The decline in paclitaxel production in older cultures is caused by age-related epigenetic changes. In addition, promoters of *GGPPS*, *TXS*, and *DBTNBT* were analyzed for their methylation patterns in a new cell line and compared with a 14-year-old cell line [67]. DNA methylation was observed in the *TXS* and *DBTNBT* promoters, leading to reduction in gene expressions. *DBTNBT* promoter is methylated before the transcription start site for both new and old cultures, but not for *TXS* and *GGPPS* promoters. This infers that latter stages of paclitaxel biosynthesis are governed by differential methylation patterns [67]. They noted that primary metabolism is preserved in long-term plant cell cultures, while secondary metabolite accumulation is lost over time. However, the addition of 5-azacytidine to *Taxus x media* cell cultures restored the paclitaxel production [81]. In *Taxus chinensis*, interaction between *miR5298b* and its target *TcNPR3* (a salicylic acid receptor homolog) was confirmed using overexpression, miRNA mimics, and downstream quantitative assays. *miR5298b* cleaves the *TcNPR3* mRNA, leading to the disruption of *TcNPR3*-*TcTGA6* corepressor complex, which alleviates the repression of taxol biosynthesis genes [82]. *miR164/taxane 13α-hydroxylase* (*T13OH*) was discovered via high-throughput degradome sequencing and small RNA sequencing. These studies identified *T13OH* and *taxane 2a-O-benzoyltransferase* (*TBT*) as the cleavage targets of *miR164* and *miR171* respectively [68]. The rest of the miRNAs (*miR396b*, *miR2673a*, *miR828b*, *miR2910*, and *phe-miR1438*) have been reported through high-throughput transcriptome sequencing paired with bioinformatics prediction algorithms. Methods must therefore be developed to reduce methylation events in older cultures and to maintain higher levels of secondary metabolites sustainably over a period of time in large scale cultivation of cells. miRNAs in *Taxus chinensis* have been discovered. Key genes such as *GGPPS-1*, *BAPT-2*, *T13aOH-3*, *TBT-4*, *T10bOH_like_5*, and *T2aOH-1* are targeted by 15 miRNAs, mostly in the leaf. These miRNAs also target TFs with functions in the regulation of plant growth. Overall, it appears that miRNAs function as intermediate signals balancing between paclitaxel biosynthesis and plant development [83,84]. In the case of paclitaxel, a causal relationship between DNA methylation and accumulation is demonstrated. Progressive loss of paclitaxel accumulation in the long term in vitro cell cultures was noticed with enhanced DNA methylation in the promoter regions of biosynthetic genes such as *taxadiene synthase* (*TXS*), and *DBTNBT*. However, causality has been established by treating with DNA methyltransferase inhibitors and targeted CRISPR-guided methylation/demethylation tools. These experiments reversed the transcriptional silencing and restored paclitaxel biosynthesis [80]. Additionally, by simultaneous action of both chemical inhibitors and precursor feeding of phenylalanine, a 3.5-fold increase in paclitaxel biosynthesis has been achieved [85].

Chromatin-based modifications enhanced the production of mitraphyllin, a spirooxindole alkaloid, known for its anti-cancer properties [86]. Similarly, using chromatin-modifying agents, genes in the paclitaxel biosynthetic pathway were activated and yields were substantially improved [80,87]. Chromosomal-scale reference-grade genomes of *Taxus chinensis* [88] and other species [89] enabled researchers to identify the genes involved in paclitaxel biosynthesis and mechanism of action. DNA methylation of three promoters of paclitaxel synthesis genes (*GGPPS*, *TXS*, and *DBTNBT*) were found responsible for the loss of paclitaxel accumulation in 14-year-old long-term cultures in comparison with freshly prepared cultures [67]. However, DBTNBT promoter is heavily methylated for both old and new cultures. These authors also noted that primary metabolism is preserved in long-term cultures, but secondary metabolism is gradually lost [67]. Addition of 5-aza-2-deoxycytidine to cell cultures of *Taxus media* restored the paclitaxel synthesis with a reduction in biomass [81].

### 4.6. Epigenetic Editing Using Dcas/9 in Plants

The use of catalytically inactive Cas9 (dCas9) for epigenome responses is an important shift in plant synthetic biology, and acts as a programmable DNA-binding scaffold. Upon fusing with artificial TFs or chromatin-modifying enzymes, dCas9 allows fine tuning of metabolic pathways. dCas9 can be fused to epigenetic effectors like DNA methyltransferases (Drm2) or demethylases (Tet1) to modify CpG methylation patterns at specific loci [90]. (Instead of targeting the biosynthetic pathway genes directly, researchers concentrated on modulating the metabolic flux of supporting pathways to redirect the resources to paclitaxel production using Dcas9-ntdrmcd complex. Knockdown of phenylalanine ammonialyase (Pal) and the use of chemical inhibitors such as Dcas9-suntag system, an advanced CRISPR tool designed for targeted epigenome editing, and used to turn on the genes involved in paclitaxel synthesis, with suppression of the competitive pathway resulted in a taxus cell line with 25-fold increase in paclitaxel accumulation in the methylated cell line palg1 in comparison with control [81]. This is the first use of CRISPR in taxus cell cultures [85], which opens the possibilities for gene editing and for improving paclitaxel accumulation. The study demonstrates the reversal of DNA methylation in plant secondary metabolism. importantly, identification of enzymes involved in paclitaxel and baccatin III biosynthesis [91,92] and the two pathways for paclitaxel biosynthesis provide us additional targets which might control paclitaxel biosynthesis. Pal knockdown through CRISPR-guided methylation resulted in 25-fold increase paclitaxel accumulation. Such a manipulation activates the silent or poorly expressed genes in cell suspensions. However, this has not been widely demonstrated in other medicinal plants that produce anticancer compounds or nutraceuticals.

## 5. Nutraceutical Accumulation and Their Regulation by Epigenetics

Nutraceutical molecules are bioactive compounds and derived from microbes, animals, and plants. They provide health benefits to humans. Some of the important categories of nutraceuticals derived from plants include (a) carotenoids such as astaxanthin, lycopene, lutein, zeaxanthin, and others derived from *Momordica cochinchinensis*, *Solanum lycopersicum*, *Haematococcus pluvialis*, a green alga, and others [93], (b) omega-3 polyunsaturated fatty acids synthesized in the seeds of *Linum usitatissimum* and other plants, (c) co-enzyme Q10 synthesized from *Glycine max* and unrefined vegetable oils [94], (d) polyphenols and flavonoids such as curcumin, resveratrol produced from *Curcuma* and *Vitis* respectively, catechin, epicatechin, epigallocatechin, epigallocatechin gallate, and flavonoids derived from green tea (*Camellia sinensis*) [95], (e) prebiotics such as inulin, fructooligosaccharides, and related fructans, which are rich in *Cichorium intybus*, *Helianthus tuberosus*, *Allium cepa*, etc. [96], and (f) vitamins such as ascorbic acid derived from *Phyllanthus emblica*. These compounds play vital roles in human health. Understanding their regulation is essential to improve their accumulation. They act as powerful antioxidants which are critical for protecting human body from oxidative stress and maintaining eye, heart, immune system regulation, mental well-being, gut, and skin health [97]. Regulation of some of these nutraceutical molecules by epigenetic mechanisms is described below. miRNA regulation of a few antioxidants is shown in the Table 2.

### 5.1. Carotenoids

#### 5.1.1. Astaxanthin

*Haematococcus pluvialis*, an algal member, produces the highest amount of astaxanthin and is the primary industrial and commercial source. It exhibits strong antioxidant activity and hence is an important nutraceutical. Low temperature plasma (LTP) stimulates the growth of *H. pluvialis* and accumulation of astaxanthin, which is associated with strigolactone and abscisic acid hormones [114]. These two phytohormones ramp up astaxanthin biosynthesis by upregulating the gene *CAROTENOID ISOMERASE* (*CRTISO*, the hub gene involved in carotenoid biosynthesis), which is also related to histone modifications like H3K4me3 (trimethylation of histone H3 at lysine 4) by LTP [114].

#### 5.1.2. Lutein

Though many plants produce lutein, primarily the highest accumulating species such *Brassica oleracea* variety *acephala* (kale) is discussed. Lutein acts as a reactive oxygen species (ROS) scavenger. It accumulates in the eyes and prevents retinal damage by filtering high-energy blue light radiation [115]. Both the carotenoids lutein and zeaxanthin neutralize free radicals and protect against visual disorders and cognition diseases such as age-related macular degeneration, age-related cataract, hypoxia-induced retinopathy, retinitis pigmentosa, and diabetic retinopathy [116]. Lutein is produced via the MEP pathway and its synthesis is dependent on the activities of *PHYTOENE SYNTHASE* (*PSY*), *LYCOPENE β-CYCLASE* (*LCYB*), and *LYCOPENE ε-CYCLASE* (LCYE) [101]. Environmental changes can either hyper- or hypomethylate the genes involved in the biosynthetic pathway in *B. oleracea* [117]. Histone modifications, especially H3K27me3, modulate gene expression to suppress or promote lutein biosynthesis [118]. In *B. oleracea* varieties *acephala*, broccoli, cabbage, and cauliflower, exposure to far-red light or shade avoidance and UV-B radiation caused epigenetic changes and triggered lutein accumulation [119]. Lutein accumulation is also developmentally regulated in kale. The highest levels of lutein in *B. oleracea* variety *acephala* were observed in mature (2–3-week-old) leaves, following a quadratic trend [120]. In kale, miR156 and miR828 suppress negative transcription factors which allow upregulation of structural genes involved in lutein biosynthesis [121]. The results reveal that miRNA network can lead to the development of climate-resilient kale plants with elevated lutein levels. In case of *Litchi chinensis*, *Lc-miR408a/b/c/d/f*, *Lc-miR172h*, and *Lc-miR160c/d* miRNAs target *carotenoid cleavage dioxygenase* (*CCD*) genes and suppress them, thus helping to accumulate valuable pigments [122].

#### 5.1.3. Lycopene from Tomato

In *Solanum lycopersicum* (tomato), carotenoid biosynthesis is dictated by DNA demethylation, resulting in varied fruit colors and nutritional value depending on the amount of lycopene accumulation [123]. Accumulation of lycopene is influenced by environmental factors (salt, drought, temperature, nanoplastic contamination, and other stresses) and the stage of fruit development. Abiotic stresses inhibit photosynthesis and stimulate *LYCOPENE β-CYCLASE* (*LYCB*), *PHYTOENE DESATURASE* (*PDS*), *PHYTOENE SYNTHASE* (*PSY1*), and ZETA-CAROTENE DESATURASE (ZDS) genes that enhance lycopene content depending on the developmental stage of fruits [124,125]. Among the environmental factors, red light irradiation promotes the synthesis of lycopene in tomato fruits, but far-red light reverses this effect [126]. Chilling stress suppresses *DEMETER-like* (*SlDML2*) gene expression, which prevents hypomethylation resulting in low lycopene accumulation [127,128]. Treating harvested fruits of tomato with 1-methylcyclopropene enhances DNA methylation of ethylene-signaling genes such as *ETHYLENE INSENSITIVE 3* (*LeEIN3*), suppressing ethylene biosynthesis and lycopene accumulation. In contrast, exogenous application of melatonin reverses this process by demethylating *AMINOCYCLOPROPANE-1-CARBOXYLASE SYNTHASE 10* (*SlACS10*) gene with a spike in lycopene accumulation [128]. During fruit ripening, demethylation occurs in *PSY1*, *PDS*, and other genes affecting the synthesis and surge of lycopene production [129]. On the other hand, in mutants like *Colourless non-ripening* (*cnr*), hypermethylation in the *SBP-box* transcription factor SPL-CNR inhibits gene expressions leading to less lycopene accumulation [130]. Silencing histone deacetylases such as *HISTONE DEACETYLASE 1* (*SlHDA1*), *SlHDA3*, etc., results in quick fruit ripening along with altered lycopene synthesis [125,129]. Many miRNAs and lncRNAs affect lycopene content by controlling the mRNA stability and translational efficiency of genes implicated in carotenoid synthesis [129]. While DNA demethylation via the *CNR* and *NON RIPENING* (*NOR*) promoters causes tomato fruit ripening and lycopene accumulation, the complex crosstalk between multiple modifiers such as DNA methylation, histone modification, and RNA methylation on lycopene-producing genes is poorly understood.

#### 5.1.4. Lycopene from *Momordica*

*Momordica cochinchinensis* is a rich source of lycopene and, hence, an important nutraceutical. Its concentrations range from 0.27 mg/g to 7.76 mg/g tissue depending on harvest site, environmental stress, and seasonal variations. Environmental cues are known to alter methylation states of DNA. During ripening of lycopene-rich fruits, demethylation occurs on a genome-wide scale, which removes methyl groups and activates specific genes [131]. Hypomethylation of pathway genes such as *PHYTOENE SYNTHASE 1* (*PSY1*) and *PHYTOENE DESATURASE* (*PDS*) allows transcriptional activity and activation of the methylerythritol 4-phosphate (MEP) pathway leading to synthesis of lycopene [123]. DNA methylation controls fruit ripening TFs which are homologs of tomato *RIPENING INHIBITOR* (*RIN*) and *COLORLESS NON RIPENING* (*CNR*). Once these TFs are hypomethylated, they form complexes boosting carotenoid pathway genes [123]. Application of 5-azacytidine inhibits carotenoid pathway genes, which delays *M. cochinchinensis* fruit color changes and reduces total lycopene accumulation [132].

### 5.2. Omega-3 Polyunsaturated Fatty Acids

Seeds of *Linum usitatissimum* (flax) are one of the richest plant-based sources of omega-3 polyunsaturated fatty acids, driven by high expression of the *FATTY ACID DESATURASE 3* (*FAD3*) gene. FAD3 is a critical enzyme and converts linoleic acid into alpha-linolenic acid (ALA). In *L. usitatissimum*, accumulation of fatty acids occurs in plastids, where oleic acid is converted to linoleic acid and then into ALA through FAD7 and FAD8 desaturase enzymes. Endoplasmic reticulum acts as the second compartment or extraplastidial pathway and is controlled by FAD3 enzyme which enables high accumulation of ALA [133]. Both DNA methylation and histone modifications modulate omega-3 fatty acid biosynthetic pathways [134]. Transcription of FAD genes is regulated by epigenetic dynamics in response to cold and drought stress conditions [135]. While hypomethylation in the promoter sequences of *FAD3* gene boosts the gene expression resulting in higher ALA accumulation, histone acetylations such as H3K9ac alter transcriptional accessibility. Abiotic stresses trigger euchromatin loosening, which helps in the binding of TFs, effectively boosting omega-3 fatty acids like ALA synthesis [136]. Several researchers are utilizing targeted epigenome editing and breeding programs aimed at stabilizing and improving oil yields and modify fatty acid ratios for nutraceutical and industrial applications. In *L. usitatissimum*, *miRNA156a* has been identified and its overexpression in a heterologous system reduced the fatty acid content [137], indicating the key role played by *miRNA156a* in fatty acid biosynthesis in flax seeds. miRNAs typically suppress target gene expression; they work within multi-layered competing endogenous RNA networks (CeRNAs) to regulate *FAD2* and *FAD3* genes implicated in ALA biosynthesis during flax seed development [102]. Therefore, miRNA regulation is crucial for enhancing oil yields.

### 5.3. Polyphenols and Flavonoids

*Curcuma longa*, *Vitis vinifera*, and *Camellia sinensis* are some of the plants rich in polyphenols and flavonoids and are widely used as nutraceuticals. Polyphenols and flavonoids act as antioxidants and anti-inflammatory agents in humans. They reduce the risk of diabetes, heart diseases, and neurodegenerative disorders. They enhance blood vessel elasticity, lower blood pressure, and reduce LDL cholesterol oxidation, lowering the risk of atherosclerosis, heart attacks, and strokes [138]. They protect insulin-producing cells from glucose toxicity. They interrupt inflammatory pathways and mitigate inflammations [138]. Polyphenols promote cerebral blood flow, and protect brain cells from age-related degeneration and oxidative damage [139]. Polyphenols neutralize free radicals, reduce tumor cell growth, and prevent formation of new blood vessels and promote apoptosis in cancer cells [139]. Polyphenols promote the growth of beneficial gut bacteria [140]. Some of the popular plants that are rich in polyphenols and flavonoids and their epigenetic regulation are described below.

#### 5.3.1. Resveratrol

*Vitis vinifera* (grapevine) and other species produce resveratrol, a stilbenoid polyphenol molecule. Biosynthesis of resveratrol is regulated by DNA methylation and histone modifications. Epigenetic dynamics regulate the *STILBENE SYNTHASE* (*STS*) gene family, thereby determining the plant’s ability to synthesize resveratrol in response to biotic and abiotic stress conditions [141]. DNA methylations reduced the resveratrol content in *Vitis amurensis* suspension cultures [142]. Methylation in promoter regions of *STS* genes silences the genes leading to lower accumulation of resveratrol. When grape cells or field-grown plants are exposed to stressors like UV irradiation or high light stress, hypomethylation occurs, allowing high expression of *STS* genes with a surge in the synthesis of resveratrol. Tyunin et al. [142] studied the role of 5-azacytidine-induced DNA demethylation on methyltransferase gene expression and accumulation of resveratrol in suspension cultures of *Vitis amurensis*. Transformation of cells with *rolB* gene showed downregulation of methyltransferase and chromomethylase 3 enzymes, but upregulation of domains in methylase proteins. Further, 5-azacytidine and the *rolB-*transgenic calli enhanced the expression of all methylases (but not methyltransferase) [142]. Further, Kiselev et al. [143] also recorded DNA methylations that greatly influence the accumulation of resveratrol. Stress signals cause histone acetylations which unwind the chromatin at the *STS* locus, resulting in the binding of *VvHY5* TF and synthesis of resveratrol. Abiotic stressors cause accumulation of ROS and commence histone modifications and increased *STS* transcription and resveratrol synthesis [144]. Under high light conditions, *VvHY5-BR-ENHANCED EXPRESSION 1* (*VvBEE1*) regulatory module plays a critical role in controlling *VvSTS* gene expression. While *VvHY5* activates *VvSTS* expression, *VvBEE1* suppresses it. *VvHY5* and *VvBEE1* compete for binding to *VvSTS* promoter, and the brassinosteroids (BR) regulate their interaction [144]. The findings demonstrate the interplay between light signals and BR pathways in modulating resveratrol synthesis. There is causal evidence that resveratrol and catechin act as epigenetic modulators. Evidence for their biosynthesis due to epigenetic changes and the corresponding pathway genes are however correlative [145]. Shifts in phenylpropanoid and flavonoid pathways and the expressions of pathways besides histone acetylations under environmental stress conditions occur; however, locus-specific epigenetic causality for their accumulation has not been demonstrated clearly.

#### 5.3.2. Catechin, Epicatechin, Epicatechin Gallate, Epigallocatechin, Epigallocatechin Gallate, and Gallocatechin

*Camellia sinensis* (green tea) contains several flavonoids and flavonols such as catechin (C), epicatechin (EC), epicatechin gallate (ECG), epigallocatechin (EGC), epigallocatechin gallate (EGCG), and gallocatechin (GC) that act as the most potent natural antioxidants. Biosynthesis of main taste compounds is coordinately regulated by miRNAs and hormones in tea plants [146]. Epigenetic mechanisms regulate the spatial, temporal, and stress-induced accumulation of these polyphenols. Hypermethylation represses the expressions of *ANTHOCYANIDIN REDUCTASE* (*CsANR*), *LEUCOANTHOCYANIDIN REDUCTASE* (*CsLAR*), and *FLAVONOID 3′,5′-HYDROXYLASE* (*CsF3′5′H*), thereby inhibiting the production of EC, EGC, and EGCG [147]. Young tea leaves accumulate high concentrations of catechin, but older leaves contain less polyphenols due to DNA methylation increases. However, varying light conditions, drought, and temperature change DNA hypermethylation, which leads to expression of genes involved in catechin biosynthesis [148]. Dynamic DNA methylation shifts have directly been correlated to catechin accumulation during tea leaf development. By integrating transcriptomic data with methylomic data, Chen et al. [149] demonstrated that genomic methylation levels directly control phenylpropanoid and flavonoid pathways structural genes (through methylation-related genes such as *CMT2*, *MET1*, and *DME*). Another study by Lian et al. [150] maps that catechin biosynthesis is driven by a functional division of labor between Whole-Genome Duplication (WGD, activating signals) and Tandem Repeat Duplication (TRD, methylation barriers) genes which are regulated by distinct epigenetic markings in tea leaf [150]. Genes that come from ancient whole-genome duplications inhabit in chromatin regions with minimal methylation barriers and dominated by activating epigenetic signals [150]. On the other hand, genes from TRD are constrained by DNA methylation and genetic variation barriers, with their baseline expressions. While WGD modules are responsible for the synthesis of epicatechin, gallocatechin, and epigallocatechin, TRD gene modules produce epigallocatechin gallate and epicatechin gallate [150]. Hypermethylation of core flavin-3-ol biosynthetic genes *CsANR*, *CsLAR*, and CsF3′5′H and the matching reduction of catechins is backed by multi-omics research in *C. sinensis* [149]. Metabolomic validation through GC-MS and other profiles shows that hypermethylation-driven silencing of structural genes like *CsLAR*, *CsANR*, *nerolidol synthase* (*NES*), *serine carboxypeptidase-like acyltransferases 1A* (*SCPL1A*), and *CsF3′5′H* suppresses metabolic flux down the catechin pathway [149]. Differentially methylated regions are primarily located in secondary metabolism related genes. Catechin, gallocatechin, gallocatechin gallate, and epicatechin gallate were significantly lower in older leaves, while catechin gallate and epicatechin were higher in older leaves [149]. *LAR* gene isoforms involved in catechin and gallocatechin were upregulated in older leaves. Expression levels of *anthocyanidin reductase* (*ANR*) responsible for the formation of epicatechin and epigallocatechin were lower in older leaves in comparison with tender leaves. Contents of gallocatechin gallate, epicatechin gallate, and epigallocatechin gallate were higher in tender leaves than in older leaves, which was in line with the expression data. 5mC DNA methylation is a conserved epistatic mark and involves developmental regulation, abiotic stress, and metabolite synthesis. However, the role of 5mC DNA methylations in tea plant growth and development and flavor substances are unknown. To improve the catechin contents, researchers need to identify the precise 5mC and m6A methylation landscapes covering the promoters of *CsPAL*, *CsCHS*, *CsDFR*, and *CsANR* genes. It is reported that some key genes during processing are regulated by 5mC methylation, which can explain the changes in aroma metabolites (nerolidol, lipids, and α-farnesene) and taste substances like catechins [147]. Their experiments reveal epimutation-related gene targets for future improvement of catechins. Further, functional characterization of native tea DNA demethylases such as *ROS1* homologs are necessary to understand and achieve targeted hypomethylation [151]. While *Cs-miR156-SPL-CsDFR* axis are known [151], many miRNAs require experimental validation to confirm their catechin pathway targets, and also how they modulate the balance between non-esterified catechins and ester-type catechins.

### 5.4. Ginsenosides, Steroid Glycosides and Triterpene Saponins

Ginseng is an important folk medicine in East Asian countries. Ginsenosides are a group of active steroid glycosides and triterpene saponins that occur naturally in the roots, stems, and leaves of *Panax ginseng* and other species. Ginsenosides regulate and increase immune cell activity and improve the body’s defense against infections [152]. They act as adaptogens and reduce physical and mental fatigue. They improve our cognitive performance. They decrease oxidative stress and help to alleviate inflammatory conditions. They improve coronary blood flow and protect heart tissues. Ginsenosides regulate blood glucose levels (antidiabetic effects) and induce apoptosis in cancer cells [152]. The production of ginsenosides varies in the field-grown plants depending on environmental stressors and developmental stages, which is driven by epigenetic changes [153]. Epigenetic pathways contribute to phenotypic plasticity of cells [154]. In American ginseng (*P. quinquefolius*) and Asian ginseng (*P. ginseng*), seasonal temperatures modulate cyclically reversible DNA methylation and demethylation [155]. Prolonged cold weather in winter triggers DNA demethylations in tender leaves of early spring. Such a localized demethylation triggers the expression of specific squalene synthase genes and oxidosqualene cyclase family members such as *dammarenediol II synthase* (*PqDDS*) which channel the carbon flux into the triterpenoid saponin framework [152,155]. However, evidence for the accumulation of ginsenosides by epigenetic changes is correlative. Studies indicate that cold-induced demethylation synchronizes with the higher expression of ginsenoside biosynthetic pathway gene *dammarenediol-II synthase* (*PqDDS*) [155]. However, this is a coordinated response, but not a direct cause and effect. Natural DNA methylations were discovered in *Panax*, which drive the seasonal accumulation, however, direct application of CRISPR-mediated, targeted epigenome demthylation has not yet been reported.

Ginsenoside biosynthesis primarily occurs in leaves but they are transported to roots, where they are stored. Conversely, if plants are not exposed to adequate cold stress, DNA hypermethylation can occur which inhibits ginsenoside biosynthetic pathways. Production of ginsenosides in cell suspensions of *Panax* are controlled by DNA methylations of promoter regions of genes such as *farnesyl pyrophosphate synthase* (*FPS*), *squalene synthase* (*SS*), and *squalene epoxidase* (*SE*) [156]. When the genes are methylated, production of ginsenosides decreases. However, treating *Panax* suspensions with methyl jasmonate decreases this methylation, leading to increased accumulation of ginsenosides [155,157]. Small ncRNAs bind to target mRNAs and degrades mRNA. For example, miR156 negatively modulates TFs involved in hormone signaling and suppress ginsenoside synthesis. LncRNAs bind to miRNAs and prevent them from silencing mRNAs implicated in biosynthesis pathway [158].

### 5.5. Inulin, Fructo-Oligosaccharides and Related Fructans (Pre-Biotics)

Plants such as *Helianthus tuberosus*, *Allium cepa*, *Allium sativum*, and *Asparagus racemosus* are the richest sources of fibers such as inulin, fructooligosaccharides, and related fructans (storage carbohydrates) which act as potent prebiotics. Human digestive enzymes cannot break them down, so fructans reach the large intestine and nourish the gut microbiome there. Fructans are fermented in the intestine by beneficial saccharolytic bacteria like *Bifidobacterium*, *Lactobacillus*, and *Anaerostipes* [159]. While the microbes consume fructans, they produce important short-chain fatty acids (SCFAs) like acetate, butyrate, and propionate [159]. Butyrate is a crucial energy source for colon cells and nourishes the intestinal lining and prevents the translocation of harmful endotoxins into the bloodstream. The microbial fermentation triggers significant physiological benefits to humans. Further, SCFAs interact with gut-associated lymphoid tissue (GALT), and prevents systemic inflammation and balances immune responses. SCFAs, once they enter the bloodstream, regulate blood sugar levels, improve insulin sensitivity, and decrease hepatic fat production [159]. Two enzymes, namely sucrose: sucrose 1-fructosyltransferase (1-SST) and fructan: fructan 1-fructosyltransferase (1-FFT), catalyze the synthesis of inulin from 1-kestose. Accumulation of inulin and fructooligosaccharides is driven by genes such as *SUCROSE-FRUCTOSE TRANSFERASES* (*1-SST* and *1-FFT*). However, no empirical evidence exits demonstrating methylation of 1-SST and 1-FFT promoters directly cause the reduction in inulin. Environmental stresses can alter the methylation patterns, affecting photosynthesis and allocation of carbohydrates while upregulating stress-response genes and inulin biosynthesis, since inulin acts as a compatible solute besides acting as an antioxidant [111,160,161]. Under cold or drought stress conditions, enzymes breakdown long-chain inulin into shorter molecules, altering the osmotic potential of the cells without wasting carbohydrates [162]. In *H. tuberosus*, the histone marks like H3K4me3 are concentrated at the transcription start site and near the 5′ end of the transcribed genes [163]. Another epigenetic mark, H3K27ac, is a known indicator of actively transcribed promoters and enhancers. This process facilitates transcription of specific genes like *1-SST* and accumulation of inulin [164]. Epigenetic regulation of inulin biosynthesis appears similar in *Allium cepa* and *A. sativum*.

### 5.6. Vitamin C or Ascorbic Acid

Vitamin C (ascorbic acid) is an essential nutrient for human health. It is an excellent antioxidant, protects cells from damage, helps in synthesizing collagen and neurotransmitters, and increases absorption of non-heme iron from plants. *Phyllanthus emblica* is a known source of high levels of vitamin C. The plant contains gallic acid which is known to mediate histone modifications in other biological systems [165]. There is evidence that demonstrates epigenetic events regulate vitamin C biosynthesis genes in *P. emblica*. However, 5-azacytidine was used to induce genome-wide hypomethylation in *Rosa roxburghii*, which produces fruits that are highly rich in vitamin C [166]. The treatment caused promoter demethylation. Whole-Genome Bisulfite Sequencing (WGBS) and RNA-seq showed that this demethylation triggered the transcriptional upregulation of vitamin C biosynthesis genes, specifically *L-galactose dehydrogenase* (*RrGalDH2*) and myo-inositol monophosphatase-like protein (*RrIMPL2*), which corresponded with significantly enhanced ascorbic acid content [166].

### 5.7. Functional Confirmation of ceRNA/ETM Mechanism

ceRNA/ETM: following are some of the functionally validated studies and confirm competing endogenous RNA (ceRNA)/endogenous target mimic (ETM), (ceRNA/ETM) mechanism using overexpression/RNAi, transient reporter assays, target gene expression rescue and metabolite quantification [167].

(a). Validated studies: It was demonstrated that the lncRNA nta-eTMX27 acts as a decoy sponge for nta-miRX27. By sequestering the miRNA, the lncRNA switching the expression of *quinolinate phosphoribosyltransferase 2* (*QPT2*), thereby driving the nicotine alkaloid biosynthesis [168]. Overexpression of lncRNA MSTRG.47662.1 sponged mes-miR398, resulting in the accumulation of the targeted transcript, and enhanced the chlorogenic acid content in tobacco and sweet potato callus lines. Conversely, direct miRNA mes-miR398 overexpression decreased the chlorogenic acid content [169]. Regression analysis of the expression patterns revealed that MSTRG.47662.1, acting as a ceRNA, boosted itb04g00990 expression by binding mes-miR398 in chlorogenic acid biosynthesis in sweet potato [169]. These results demonstrate insights into how ncRNA-mediated ceRNA regulatory networks contribute to secondary metabolite synthesis.

(b). Prediction and correlation-based studies: These studies establish exploratory candidate networks; however, genetic rescue lines or biochemical binding confirmation are lacking, thereby demonstrating that the lncRNA physically competes to modify the secondary metabolite accumulation. While comparing high- and low-artemisinin producing chemotypes, Ma et al. [170] discovered three potential lncRNAs (MSTRG.33718.2, MSTRG.30396.1, andMSTRG.2697.4) correlating with *amorpha-4,11-diene synthase* (*ADS*), *1-deoxy-D-xylulose 5-phosphate synthase* (*DXS*), and *3-hydroxyl-3-methylglutaryl CoA synthase* (*HMGS*) involved in artemisinin biosynthesis. They noticed upregulation of these lncRNAs positively modulate the target artemisinin pathway genes, resulting in high accumulation in high artemisinin-producing lines. Interestingly, *β-amyrin synthase* (*BAS*) gene involved in the artemisinin competing pathway was downregulated in these plants in comparison with low artemisinin-producing lines, which correlates well with expression patterns of the linked lncRNA MSTRG.30396.1 [170]. These novel findings pave the way for metabolic engineering and for increasing the valuable secondary metabolite accumulation. Transcriptomic survey construct complex lncRNA-mRNA-TF co-expression networks and link non-coding transcripts to phenylpropanoid or terpenoid (salvianolic acid and tanshinone) accumulation in *Salvia miltiorrhiza* [171]. Combining co-expression and genomic location analysis, Wang et al. [171] obtained 23 candidate lncRNA-mRNA/TF pairs that were co-expressed and co-located. They also analyzed the time-series expression of *S. miltiorrhiza* induced by MeJA [171]. They noticed that four lncRNAs, two mRNAs, and two TFs formed three lncRNA-mRNA and/or TF network modules. Their regulation might help us to manipulate diterpenoid biosynthetic pathway of *S. miltiorrhiza*.

### 5.8. Differentiation Between an Increase in Metabolite Concentration and an Increase in Industrial Productivity per Volume and Time

Often we fail to distinguish between metabolite concentration and industrial productivity leading to engineering strategies that look successful in the lab, however, fail on a commercial scale. this distinction is critical while dealing with growth-defense and trade-offs. For example, elicitors or nutrient deprivations in the medium boosts the secondary metabolite levels, while simultaneously shutting down the cell or plant biomass accumulation [172]. This problem persists in majority of cultured plant cells and even at the whole plant level. it is vital therefore for us to balance the plant growth versus the secondary metabolite levels. Upon applying the stress, or when we divert the primary metabolism to secondary metabolism, metabolite concentration per cell increases. However, such a strategy if limits plant growth or cell biomass, the industrial productivity per definite volume and specific time totally collapses and the industrial scale production is hampered [173]. For example, both biotic and abiotic elicitors increase the concentration of artemisinin (mg/g DW). When the stresses reduce or stop the growth of cell suspensions or plants, harvestable biomass per square meter per month decreases, which makes the process non-viable commercially [174]. In industrial bioreactors, adding MeJA to the suspension cultures of taxus curbs the cell cycle progression and biomass. If the elicitor is added too early, secondary paclitaxel content increases, but the volumetric productivity (mg/L/day) remains low due to minimal cell density in the reactor [175]. To overcome the classic trade-off or limitation, industrialists grow plant cell first to a high density in a growth-optimized medium, and then treat the biomass with MeJA or transfer biomass to a production medium to spike paclitaxel synthesis without any decrease in biomass [176].

## 6. Transversal Discussion Conclusions

After an integrated evaluation of diverse anticancer compounds and nutraceuticals, the following discussion provides some points for understanding and for future research directions. Currently, the major transversal trend is the divergence between callus or suspension cultures which are not organized and highly organized and differentiated tissues like hairy root and shoot cultures. In the case of *Taxus x media* long-term suspensions, epigenetic drift occurs, especially DNA hypermethylation, which targets selectively pathway promoters at specific loci. Hypermethylation is primarily targeted in the promoter sequences of the gene *taxadiene synthase* (*TXS*) in *Taxus*. For indole alkaloids like vincristine in *Catharanthus roseus*, the mechanism mostly depends on chromatin accessibility which is linked to tissue differentiation. In wild plant leaf tissues, open chromatin configurations allow multi-compartmentalized pathway transcription. On the other hand, in suspensions localized chromatin compaction occurs which preclude necessary TF binding. This suggests that callus versus intact organs dictates the active epigenomic landscape. However, the reversibility of epigenetic modifications varies in secondary metabolic pathways depending on the species. Histone deacetylase inhibitors like suberoylanilide hydroxamic acid (SAHA) and DNA methytransferase inhibitors such as 5-azacytidine exhibit transient reversibility epigenetic modifications. In cell cultures, time-dependent epigenetic changes occur in *Taxus* and *Beta vulgaris*. Treating such cultures with demethylating agents gives varying levels of success [80,81]. These results reveal that in specific plants, long-term cultures make certain hypermethylated loci which are resistant to reversibility or restoration. Early studies depended on profiling correlation-matching global/locus-specific methylation signatures (correlation evidence) with a decline or rise in secondary metabolite levels. Adding azacytidine or nicotinamide to the medium to stimulate phenotypic changes besides substantiating that altering epigenome alters metabolic output (functional evidence). An important tier of evidence for direct genetic causality has been shown through CRISPR/dCas9-based epigenome editing in plants. Such a precise modification links single epigenetic regulators to the expression of biosynthetic pathway gene clusters without any disturbance in the core genome sequence. Unlike in microbial systems (where biosynthetic gene clusters are detected), in plants, the paclitaxel or vincristine pathways are scattered across the genome. As a result, epigenetic manipulation in plants seldom acts like an “on/off” switch. Furthermore, it modifies productivity by upregulating rate-limiting enzymes while suppressing other enzymes.

In *Eleutherococcus senticosus* (Siberian ginseng), moderate drought decreased DNA methylation throughout the genome and at the promoters of *EsFPS*, *EsSS*, and *EsSE*. TFs such as *EsMYB-r1*, which was suppressed by DNA methylation has been found to re-bind to the *EsFPS* promoter following DNA demethylation, leading to saponin accumulation [28].

In tomato, microRNAs such as *slymiR-159a* act as negative regulators of L-galactose biosynthesis pathway genes like GDP-D-mannose 3,5-epimerase (GME), causing a decline in vitamin synthesis. A knockdown of this microRNA is highly useful to improve vitamin C [177]. Here, the scope exists to suppress microRNAs using both epigenetic and CRISPR/Cas9-based genome editing techniques and to enhance vitamin C in tomato and other plants.

In *Cichorium intybus*, treating plants with 5-azacytidine acts as a negative regulator and induces early flowering via DNA hypomethylation, shortening the plant vegetative phase to flowering phase, thereby preventing plant to synthesize and store inulin [178]. Therefore, one way to improve inulin is overexpression of fructan biosynthetic pathway genes such as *1-SST*, *1-FFT* in the plant. Since 5-azacytidine decreases inulin, promoting hypermethylated state on repressors of inulin biosynthesis can help us. Regulating DNA methyltransferases (MET1 and CMT3) or histones via molecular methods can keep the chromatins hosting the *1-SST* and *1-FFT* genes open to produce higher inulin levels.

The development of next-generation deep-sequencing technologies and associated bioinformatics tools have accelerated the discovery and functional validation of various epigenetic targets including ncRNAs, and their functional validation needs to be tapped into to engineer medicinal plants or cell culture optimization to meet industry standards. Importantly, it is encouraging to note that lncRNAs and circRNAs have been discovered which target and modulate multiple secondary plant product biosynthetic pathways and the accumulation of the compounds. However, several challenges remain unattended, but needs tangible and pertinent solutions. Experimental protocols to validate the function of miRNAs, siRNAs, lncRNAs, and circRNAs using genome editing and other biotechnical applications needs to be fully explored for large-scale validation to create industrial applications.

To successfully regulate epigenetic changes for producing high-value anticancer and nutraceutical compounds of commercial interest optimally, future research should focus on functional profiling. Using methyltransferase inhibitors leads to global changes in the genome across the plant; therefore, we need to develop programmable, locus-specific, epigenetic editing tools such as dCas9-Sun-Tag fused with specific methyltransferases or acetyl transferases to target pathway gene clusters of the target metabolite. We lack currently a functional directory matching specific histone modifications or DNA methylations to the target secondary metabolite. Therefore, it is necessary to establish multi-omic screening pipelines that combines single-cell ChIP-seq/Bisulfite-seq with high resolution LC-MS/MS, which can pinpoint the exact chromatin states that regulate the metabolic pathways. Environmental stressors trigger the accumulation of anticancer and nutraceutical compounds; however, this response is transient and significantly variable. Research must focus, therefore, on stabilizing epigenetic memory, which can enable inheritance of high-yielding states across generations. When complex plant pathways are transferred into microbial systems, host organism transcriptionally silences the foreign genes via native epigenetic system. Hence, we should engineer the host-genome to prevent it from silencing foreign DNA.

## Figures and Tables

**Table 1:** Role of epigenetic and post-transcriptional gatekeepers for the accumulation of anticancer compounds.

Plant Name	Field-Grown Plant/Callus/ Suspensions	Type of Methylation	Target Compound	Reference
*Taxus* sps.	Cell cultures	Methylation of this specific region leads to loss of gene expression, often observed in older cell lines that lose their capacity to produce paclitaxel	Methylation decreased the paclitaxel accumulation. Reducing methylation by CRISPR-guided tools to target PAL increased paclitaxel accumulation up to 25-fold	[66]
*Taxus* sps.	Whole plants	DNA methylation sites discovered	*TAXADIENE SYNTHASE* (*TXS*) and 3′-N-DEBENZOYL-2′-*DEOXYTAXOL-N-BENZOYL TRANSFERASE* (*DBTNBT*) promoters have been found methylated	[67]
*Taxus mairei*	Leaves	Predicted that miR164 targets taxane 13α-hydroxylase, and acts as a negative regulator of taxol biosynthesis. miR171 taxane 2α-*O*-benzoyltransferases (TBT)	Dual-luciferase assays showed that *MYB1L* activated the expression of *TBT* and *BACCATIN III 13-O-*(*3-AMINO-3-PHENYLPROPANOYL*) *TRANSFERASE* (*BAPT*). The data indicate that *miR858b-MYB1L* module plays a crucial role in taxol biosynthesis by up-regulating the expression of *TBT* and *BAPT* genes in the endodermis	[68,69]
*Catharanthus roseus*	Whole plants	*miR160* targets the *AUXIN RESPONSE FACTOR*S *CrARF10* and *CrARF16* affecting the expression of *tryptophan decarboxylase* (*TDC*), *strictosidine synthase* (*STR*) and *geraniol 10-hydroxylase* (*G10H*); *miR5021* targets *(E)-4-hydroxy-3-methyl-but-2-enyl diphosphate synthase* (*GCPE*) to modulate vincristine and vinblastine	Decreased vincristine and vinblastine accumulation	[70,71]
*C. roseus*	Whole plant	*miR156a*/*SQUAMOSA Promoter-Binding Protein-Like* (*SPL*) module regulate alkaloid biosynthesis	Inhibition of activity of *CrSPL2/3/4/5* TFs and terpenoid indole alkaloid synthesis	[72]
*C. roseus*	Whole plant	*Crlnc440* exhibited conservation among species within Apocynaceae. LncRNAs exhibited organ-specific expression and responded to MeJA	*Crlnc440* is negatively associated with monoterpene indole alkaloid biosynthesis. Its transient overexpression in *C. roseus* leaves led to significant reductions in vindoline (23%), vinblastine (33%). *Crlnc343* and *Crlnc1096* were positively associated whit MIA biosynthesis. Overexpression of *Crlnc343* resulted in significant increases in vinblastine (142%), anhydrovinblastine (175%), and catharanthine (64.5%). *Crlnc1096* over-expression elevated vinblastine (151%), anhydrovinblastine (35%), and catharanthine (30%)	[73]
*Nothapodytes nimmoniana*, *Miquelia dentata*	Whole plants along with attenuated fungi *Phomopsis* sp.	Treated with 5-azacytidine (hypomethylation)	Attenuated endophytic fungi produced 71.83 ± 7.36 µg podophyllotoxin per 100 mg of tissue. When reintroduced into *Nothapodytes nimmoniana* and *Miquelia dentata* produced 197.71 ± 10.05 µg of camptothecin per 100 mg of tissue. Attenuated fungus when cultured with 5-azacytidine enhanced camptothecin from 36 µg to 88 µg/100 mg	[56]
*Podophyllum hexandrum*	Whole plant	MeJA upregulated podophyllotoxin biosynthetic pathway genes (non-responsive to ROS) which are controlled by miR172i, miR035, miR1438, miR2275, and miR8291	Increased podophyllotoxin by two possible ways, one through increasing the mRNA stability of *PhCAD*3, *PhCAD*4, and *NAC*3 by MeJA induced ROS and another through down regulating miRNAs which are targeted to other important genes of the podophyllotoxin biosynthetic pathway	[74]
*Sino-* *podophyllum hexandrum*	Whole plants	While *miR396b* targets UDP-glycosyltransferases, *miR2673a* targets *WRKY37*, *MYBF1*, *miR828b* targets phosphoenolpyruvate carboxylase, *miR2910* targets malate dehydrogenase, and phe-miR1438 targets caffeoyl-CoA *O*-methyltransferase to regulate podophyllotoxin synthesis	*miR396b*, *miR2673a*, *miR828b* and *miR2910* established negative correlation with their mRNA targets, so decrease the accumulation of podophyllotoxin	[75]
*Linum album*	Cell cultures	MeJA mediates podophyllotoxin biosynthesis in *L*. *album* cells by inducing H_2_O_2_ and SA formation, which upregulate the expression level of *miR156*, *miR159*, *miR172*, and *miR396* and biosynthetic genes in a redox balance-dependent manner	Improved podophyllotoxin	[76]

**Table 2:** miRNAs/long non-coding RNAs-mediated regulation of nutraceuticals.

Plant Name and Ingredient	miRNAs or Long Non-Coding RNAs and Mutants Generated	LncRNAs or Target Genes of miRNAs	Target Compound	Reference
*Haematococcus pluvialis* (Astaxanthin)	No mutants	Eighty-three high light stress-responsive, 46 sodium acetate-responsive, and 14 to both light and sodium acetate stress-responsive microRNAs were detected in *Haematococcus pluvialis*	Results indicate regulatory mechanisms of miRNAs responding to high light and sodium acetate in the alga, and their modulation in astaxanthin synthesis	[98]
*Solanum lycopersicum* (Lycopene)	Silencing the lncRNA1840	Silencing the lncRNA retards autolytic surge of ethylene hormone	Suppression of ethylene synthesis delays fruit ripening in wild-type tomatoes and decreases lycopene content	[99]
*S. lycopersicum* (Lycopene)	CRISPR/Cas9-mediated loss of function mutants	lncRNA1459	Delayed fruit ripening, reduced ethylene production, and repressed lycopene levels in comparison with wild-type tomatoes	[100]
*S. lycopersicum* (Lycopene)	Knock out mutants	lncRNA1471	Accelerated ripening and significant deeper coloration with improved levels of lycopene content	[101]
*S. lycopersicum* (Lycopene)	Direct downstream target of the ripening-related Ripening Inhibitor (RIN) MADS-box TF	lncRNA2155 does target a single structural gene directly, however, it acts as a downstream, and targets the Ripening Inhibitor (RIN) MADS-box TF	By acting as a direct downstream target of the ripening-related TF RIN MADS-box, the lncRNA2155 acts as a negative regulator of fruit ripening and responsible for decreased carotenoid/lycopene content	[101]
*Linum usitatissmum* (Omega-3-polyunsaturated fatty acids)	Lnc *MSTRG.20631.1* could act as a competing endogenous RNA (ceRNA) with *miR156* and target the same gene	Lnc RNA *MSTRG.20631.1* and *miRNA156* module target squamosa promoter-binding-like protein (*SPL*) gene	Lnc RNA *MSTRG.20631.1* could act with *miR156* on the same target, SPL, to influence fatty acid biosynthesis through a gluconeogenesis-related pathway during flax seed development	[102]
*Brassica* (Omega-3-polyunsaturated fatty acids)	*miR172*, *miR395*	These miRNAs target *B-ketoacyl-ACP synthase1* (*KAS1*) and *diacylglycerol acetyltransferase* (*DGAT*)	By repressing KAS1 and DGAT genes, fatty acid synthesis is restricted for conversion into omega-3-fatty acids. Under abiotic stress conditions ABA accumulates, which downregulates miR172 and miR395, de-repressing *KAS1* and *DGAT* genes and to enhance fatty acid synthesis	[103]
*Curcuma longa* (Curcumin)	*miR1168.2, miR156b* and *miR1858*	*miR1168.2* targets *trans-cinnamate 4-monooxygenase* (*CYP73A*) gene, which catalyzes trans-cinnamate to p-coumaroyl-CoA. *miR156b* targets the *4-coumarate-CoA ligase* (*4CL*) gene, which helps in the biosynthesis of p-coumaroyl-CoA and related CoA esters. *miR1858* also targets *4CL* gene and work in concert to regulate the pathway	*miR1168.2*, miR156b and miR1858 inhibit *CYP73A*, and *4CL* in curcumin biosynthesis and also accumulation of curcumin. However, no information is available regarding lncRNAs	[104]
*Camellia chinensis*	*csn-miR2593e* and *csn-miR167a*	Regulation of enzymes in the flavonoid biosynthetic pathway including dihydroflavonol 4-reductase (DFR)	*csn-miR2593e* and *csn-miR167a* act as negative regulators of the flavonoid pathway. High expression of these miRNAs inhibit the synthesis of catechins, epicatechins and epigallocatechins	[105]
*C. chinensis*	*csn-miR4380a* and *csn-miR3444b*	*csn-miR4380a* targets structural genes involved in the catechin biosynthetic pathway such as *dihydroflavonol 4-reductase* (*DFR*) and *anthocyanidin reductase* (*ANR*), and downregulates them. Additionally, *csn-miR3444b* targets *MYB* TFs that govern *leucoanthocyanidin reductase* (*LAR*), and *anthocyanidin reductase* (*ANR*)	Downregulation of structural genes *DFR*, and the TF *MYB* that regulates *LAR* and *ANR*, limit the precursors for the synthesis of catechin and epicatechin. *miR344b* inhibits the transcription of the enzymes involved for converting leucoanthocyanidins and anthocyanidins into catechins, epicatechins, and epigallocatechins	[105]
*Camellia chinensis*	*miR529d* *miR156g-3p*	While *miR529d* targets the *CHALCONE ISOMERASE* (*CHI*), *miR156g-3p* targets *FLAVANONE 3-HYDROXYLASE* (*F3H*) gene	miRNAs downregulate the expression of *CHI* and *F3H* genes, thereby reduce total and gallated catechin levels	[106]
*C. chinensis*	*miR319h*	*miR319h* targets *Teosinte Branched/Cycloidea/PCFI* (*TCP*) TFs, like *CsTCP3* and *CsTCP4* which trigger structural genes involved in catechin synthesis	*CsTCP3*, and *CsTCP4* act directly or indirectly as positive regulators of catechin biosynthetic structural genes like *LEUCOANTHOCYANIDIN REDUCTASE* (*LARb*) and *ANTHOCYANIDIN REDUCTASE* (*ANRa*, *ANRb*) which improve epicatechin and epigallocatechin levels	[107]
*Panax ginseng* (Ginsenosides/ triterpenoids)	*pgi-novel-1899* miRNA	Two *FARNESYL PYROPHOSPHATE SYNTHASE* (*PgFPPS*) genes with ginseng-specific *pgi-novel-1899* miRNA, targeting *FPPS* genes	*pgi-novel-1899* miRNA, once targets *FPPS* genes, accumulation of ginsenoside reduces in hairy roots or in whole plants. Higher expression of *FPPS* lead to 2.4-fold increase in ginsenoside content in hairy roots	[108,109,110]
*Helianthus tuberosus* (inulin and fructo-oligo-saccharides)	*miR390-TAS3-ARF* pathway and *miR858*	*miR390-TAS-ARF* pathway and *miR858* are regulatory microRNAs but not directly involved in inulin synthesis. However, they regulate plant growth, root development, cold and drought stress responses, auxin signaling, and down stream responses	Low auxin levels upregulate *SUCROSE: SUCROSE 1-FRUCTOSYL-TRANSFERASE* (*1-SST*) and *FRUCTAN: FRUCTAN 1-FRUCTOSYL-* *TRANSFERASE* (*1-FFT*), and increases inulin accumulation	[111]
*Phyllanthus emblica* (Vitamin C)	miR156 and miR396	miR156 and miR396 control the upstream genetic switches, their target genes like *SQUAMOSA PROMOTER BINDING PROTEIN-LIKE* (SPL), and *GROWTH-REGULATING FACTOR* (GRF) dictate the flux through the main biosynthetic routes	Improved ascorbatic acid content	[112,113]

## Data Availability

No new data were created or analyzed in this study. Data sharing is not applicable to this article.
